# First Molecular Diagnosis of Clinical Cases of Gastric Anisakiosis in Spain

**DOI:** 10.3390/genes11040452

**Published:** 2020-04-22

**Authors:** Xavier Roca-Geronès, M. Magdalena Alcover, Carla Godínez-González, Olga González-Moreno, Miquel Masachs, Roser Fisa, Isabel Montoliu

**Affiliations:** 1 Laboratory of Parasitology, Department of Biology, Health and Environment, Faculty of Pharmacy and Food Sciences, University of Barcelona, 08028 Barcelona, Spain; xevirg@gmail.com (X.R.-G.); mmagdalenaalcoveramengual@ub.edu (M.M.A.); carla8610@gmail.com (C.G.-G.); olgagonzalezmoreno@ub.edu (O.G.-M.); rfisa@ub.edu (R.F.); 2 Department of Microbiology and Parasitology, SYNLAB Global Diagnostics, Esplugues de Llobregat, 08950 Barcelona, Spain; 3 Digestive Endoscopy Service, University Hospital Vall d’Hebron, 08035 Barcelona, Spain; miquelmasachs@gmail.com

**Keywords:** *Anisakis simplex* (s.s.), hake consumption, PCR RFLP, EF1 α-1 sequencing

## Abstract

Anisakiosis is a fish-borne disease with gastrointestinal and/or allergic symptoms caused by the consumption of raw or undercooked fish parasitized with nematode larvae of the genus *Anisakis*. In Europe, *Anisakis pegreffii* has been detected as the causative agent, although the sibling species *Anisakis simplex* sensu stricto (s.s.) is also known to cause the disease in other parts of the world, and discrepancies exist regarding their respective pathogenic potential. In Spain a high number of cases has been recorded, with marinated anchovies being the main source of infection, although no specific diagnosis has been documented in humans. In this study, we analyzed three cases of anisakiosis in patients from Barcelona (Spain) who had consumed undercooked hake. All patients described epigastric pain and several larval nematodes were removed endoscopically from their stomachs. Larvae were morphologically characterized as third-stage larvae of *Anisakis simplex* sensu lato (s.l.) and molecularly identified as *A. simplex* (s.s.) by means of PCR RFLP of the ITS region of the rDNA and sequencing of the elongation factor1 alpha1 (EF1 α-1) nDNA gen. This study represents the first specific identification of *Anisakis* larvae in clinical cases of anisakiosis reported in Spain. Specific molecular diagnosis is of crucial importance for assessing the health risk of *Anisakis* sibling species. Hake consumption stands out as a risk factor for anisakiosis, since this fish species can be highly parasitized.

## 1. Introduction

Anisakiosis is caused by the consumption of raw or undercooked fish and cephalopods parasitized with larvae of anisakid nematodes. The main causative agents of the disease are species of *Anisakis*, although *Pseudoterranova* and sporadically *Contracaecum* species can produce pathology, in this case known as anisakidosis [1]. Dietary habits play an important role in the transmission of anisakids, the first reported human case occurring in the Netherlands after the consumption of herring [2]. Anisakiosis is a particularly serious public health issue in Japan, associated with the tradition of eating raw fish in dishes such as sushi or sashimi. In Europe, the disease has been documented in various countries, the highest incidence occurring in Spain, where it is mainly caused by the consumption of marinated anchovies, a typical Mediterranean dish [3,4,5,6,7]. The growing incidence of anisakiosis reported in recent years, related to worldwide changes in eating habits and an increasing consumption of fish products, has led the European Food Safety Authority to rank anisakid nematodes as an important “biological hazard” in seafood products [3,5,6,8]. For the control and prevention of anisakiosis, the current Spanish legislation and European regulation require that food establishments freeze fish for at least 24 h at –20 ºC when it is to be consumed raw or marinated and recommends the same measure for dishes prepared at home [9,10]. The greater awareness of the disease, together with the development of improved diagnostic tools, has increased the number of globally documented clinical cases [11].

The most frequent forms of anisakiosis are gastric and intestinal. In these cases, third-stage larvae (L3) adhere to and penetrate the mucosal layer of the stomach or intestine, causing epigastric and/or abdominal pain and usually nausea and vomiting [11,12]. The presence of anisakid larvae, mainly *Anisakis* species, in consumed fish can also trigger allergic reactions of different degrees of pathogenicity, due to IgE-*Anisakis* hypersensitivity, the symptoms ranging from moderate, such as urticaria and angioedema, to severe, including anaphylactic shock [11]. In gastro-allergic cases, the patient develops both types of symptoms simultaneously [3,11,12]. 

The main species implicated in human anisakiosis belong to the complex *A. simplex* (s.l.): *A. simplex* (s.s.) and *A. pegreffii*. The two species have a wide defined distribution, however their range of localization overlaps in some basin waters, such as the Spanish Atlantic coast and the Japanese Sea. The third species within the *A. simplex* (s.l.) complex, *Anisakis berlandi*, which has a discontinuous distribution in the Austral region, has not been implicated in the disease [8].

In the last two decades, the use of molecular methods as PCR RFLP, DNA sequencing or, more recently DNA microsatellites, has improved the specific identification of larvae of morphologically non-differentiable *Anisakis* species, such as those of the *A. simplex* (s.l.) complex [12,13,14]. To date, it has not been established if any other *Anisakis* species can cause anisakiosis in humans [8]. Specific diagnosis of the parasite in human cases is important, not only from a taxonomical point of view, but also to understand if differences in pathology are species-related [6]. Some authors suggest that *A. simplex* (s.s.) is more resistant to gastric acid than *A. pegreffii* and has a higher rate of penetration in the stomach, small intestine or colon wall [15]. Despite widespread use of molecular techniques, in all known cases in Spain the causative agent of the disease has only been identified at the *Anisakis* genus level or as *A. simplex* (s.l.) [16,17,18,19]. 

The aim of the present study was to perform a specific diagnosis of anisakiosis and thereby shed light on the species-related health risk for humans. *Anisakis* larvae obtained from three human cases of gastric anisakiosis, caused by the consumption of undercooked hake, were characterized and for the first time in Spain the causative species were molecularly identified.

## 2. Materials and Methods

Two women aged 45 and 49 years and one man aged 21 suffering acute epigastric pain were attended in three hospitals in the Mediterranean city of Barcelona, in the North-East of Spain, in January 2019. The three patients declared they had consumed fresh undercooked hake at home within 24 h before their hospital admission. In all cases the fish was purchased in a supermarket chain, although the capture zone is not known. During the medical examination, 12, 5, and 1 nematode larvae were detected adhered to the mucosal layer of the stomach of the three patients, respectively, and in the former a thickening of the gastric antrum was observed. The larvae were endoscopically removed, and the symptoms remitted spontaneously. All nematodes were preserved in 70% alcohol, and 7 specimens from the first patient, 5 from the second and 1 from the third were available for analysis.

The morphology of the larvae was studied microscopically [20]. For molecular analysis, DNA was isolated using the Pure PCR Template Extraction Kit (Roche). The internal transcribed spacer (ITS) 1, 5.8S and ITS2 regions of the rDNA were amplified with primers NC2 and NC5 and restriction fragment length polymorphism (RFLP) was carried out using the restriction endonucleases *Hinf*I and *Hha*I [21]. The elongation factor1 alpha1 (EF1 α-1) nDNA gene was amplified and sequenced using EF-F and EF-R primers [22].

## 3. Results

**Figure 1:**
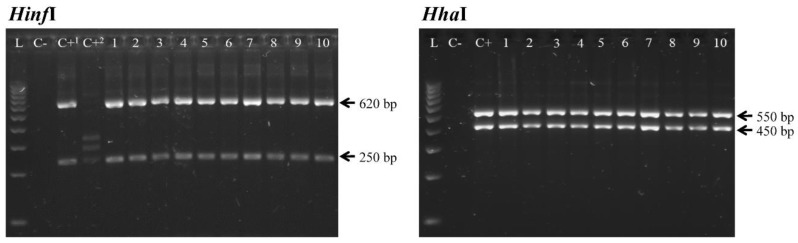
Restriction fragment length polymorphism (RFLP) patterns of *Anisakis* larvae recovered from the three patients. *Hinf*I and *Hha*I restriction enzymes of the internal transcribed spacer (ITS) region of the rDNA were used. L: 1000 bp ladder; C-: negative control; C+^1^ and C+: positive control of *A. simplex* (s.s.); C+^2^: positive control of *A. pegreffii*; lines 1–5: *A. simplex* (s.s.) from the first female patient; lines 6–9: *A. simplex* (s.s.) from the second female patient; line 10: *A. simplex* (s.s.) from the male patient.

The larvae from the three patients were morphologically identified as L3 larvae of *A. simplex* (s.l.), based mainly on the presence of a mucron at the caudal end and an elongated ventricle with an oblique posterior end. The specific patterns obtained by PCR-RFLP, using *Hinf*I and *Hha*I, showed that all the larvae studied belonged to the species *A. simplex* (s.s.), differentiating them from the other two sibling species *A. pegreffii* and *A. berlandi* (Figure 1). In addition, sequencing of the EF1 α-1 nDNA gene showed the corresponding nucleotides for *A. simplex* (s.s.) (KT825685) [22]. Sequences of the studied larvae were deposited in GenBank under the accession numbers MK905220-3.

## 4. Discussion

Several cases of gastric, intestinal and allergic anisakiosis have been reported in Spain in recent years, the first one being documented in 1991 [23]. Most of the gastric and intestinal cases are associated with the consumption of marinated anchovies, a typical dish in Spanish cuisine, although hake and sardine have also been reported as a source of infection [16,17,18,19]. The three anisakiosis cases studied here, in which female patients were infected with a relatively high number of larvae, developed after hake consumption. A variable number of larvae per patient have been detected in Spanish cases, including one particular individual with over 200 larvae in the stomach after consuming hake [18]. Nevertheless, the pathology is usually caused by a single larva [11]. The consumption of hake is also known to have triggered cases of allergic anisakiosis in Spain, mainly in the northern regions, which were diagnosed by the detection of *Anisakis*-specific IgE [11,24,25].

None of the documented clinical cases in Spain report specific identification of larvae. *A. simplex* (s.s.) has been frequently detected in fish species commonly consumed in Spain caught in the North-East Atlantic, including the Alboran Sea region, and shows only a sporadic presence in Mediterranean fishes, specifically those caught near the African coast [8]. The sibling species *A. pegreffii*, on the other hand, is endemic to the Mediterranean Sea, but has also been reported in fish caught off the North-East Atlantic coast [8,26]. Compared to Atlantic fishes, those from the Mediterranean have a lower parasitic load in terms of anisakid larvae, but both are readily available on the market [26,27]. All patients in our study live in the Mediterranean area, yet the identification of *A. simplex* (s.s.) as the causative agent suggests that the hake consumed was caught in the North-East Atlantic region. In fact, in the epidemiological studies on hake from North-East Atlantic and Mediterranean, *A. simplex* (s.s.) was not found in the Mediterranean hosts [26]. Although it can be heavily parasitized, North-East Atlantic hake is highly appreciated by consumers [27].

Elsewhere in Europe, in Italy and Croatia, only *A. pegreffii* larvae have been molecularly identified in human anisakiosis cases [12,28]. In these countries the main source of fresh marine fish for consumption is the Mediterranean Sea, where *A. pegreffii* is endemic [8]. In other parts of the world, both sibling species have been identified as responsible for the disease, *A. simplex* (s.s.) being the main agent in Japan and *A. pegreffii* in Korea [13,29]. The diagnostic disparity between geographically close countries could be explained by the notably different prevalence of parasitation of the two *Anisakis* species observed in fish between capture zones [13].

The results presented here constitute the first species-specific identification of larvae implicated in human anisakiosis in Spain. In the three studied cases all the larvae were identified as *A. simplex* (s.s.). Nevertheless, the presence of *A. pegreffii* in Mediterranean fishes widely consumed in Spain implies that this parasite species would be also involved in the disease. The health risk posed by both sibling species should be assessed by more frequent use of molecular specific diagnosis in clinical cases of anisakiosis. The results of this study also highlight hake consumption as a risk factor for the disease, as this species of fish can be heavily parasitized [27,30]. Consumers should therefore be warned of the risk of consuming undercooked fish and be better informed of the recommendations to follow.
